# Anthropometrics and Plasma Atherogenic Index in Saudi Women Madinah KSA

**DOI:** 10.12669/pjms.40.3.8318

**Published:** 2024

**Authors:** Amal M. Qasem Surrati, Walaa Mohammedsaeed, Eman Mohammed Alfadhli

**Affiliations:** 1 Amal M.Qasem Surrati, Department of Family and Community Medicine, College of Medicine, Taibah University, Medina, Saudi Arabia; 2 Walaa Mohammedsaeed, Department of Medical Laboratory Technology, Faculty of Applied Medical Science, Taibah University, Medina, Saudi Arabia; 3 Eman Mohammed Alfadhli, Professor at Medical Department, College of Medicine Taibah University, Saudi Arabia. King Faisal Specialist Hospital, Medina, Saudi Arabia

**Keywords:** Body Mass Index, Atherogenic Index of Plasma, Lipid profiles, Females, CVD

## Abstract

**Objective:**

to determine the lipid profile levels and association with anthropometric measurements and atherogenic index of plasma values in females from Taibah University.

**Methods:**

A cross-sectional study was conducted from January 2019 to January 2020 at the female section of Taibah University, located in Madinah, Saudi Arabia. The study sample consisted of 240 females ranging from 19 to 50 years. Measurements related to anthropometry such as height, weight, waist, and hip circumference, were calculated. Body Mass Index, Lipid profiles, and Atherogenic Index of Plasma were also measured.

**Results:**

Almost 73.4% of the participants were obese and overweight, with a mean BMI of 28.79±5.7 kg/m^2^. Overweight and obese women were observed to have high total cholesterol and triglyceride levels (P≤0.05). Out of 244 participants, 120 (49.2%) and 44 (18%) were at intermediate and high risk of cardiovascular disease (CVD), respectively, as determined by the atherogenic index of plasma AIP. Intermediate and high-risk CVD groups had higher lipid profile levels and high waist-to-hip ratio compared to those in females at low risk (P≤0.05). AIP was positively and significantly associated with total cholesterol and triglyceride but negatively correlated with HDL concentration. Furthermore, the BMI had significantly positive correlation with triglyceride and waist to hip ratio (P≤0.05).

**Conclusion:**

The majority of the participants were overweight and obese, with high levels of triglycerides and total cholesterol and high waist to hip ratio, placing them at intermediate or high risk of CVD based on AIP values. Additional CVD risk screenings, targeted specifically at overweight and obese women, are needed.

## INTRODUCTION

KSA vision 2030’s emphasis on preventing non-communicable diseases with a high incidence among Saudis, such as cardiovascular disease (CVD), Type-2 diabetes mellitus (T2DM), dyslipidemia, and obesity, is expected to enhance the present level of 73.3% to 96.2% by 2035.^1^,^2^ The main CVD risk factors include gender, age, body mass index (BMI), blood pressure (systolic), diabetes, hypertension, and elevated cholesterol.^1^ A Saudi study indicates that CVD risk factors are common among the Saudi population, with over half of the population being obese and over a third suffering from sedentary lifestyles and dyslipidemia.^3^

According to the most recent data from the Ministry of Health (MoH), hyperglycemia, diabetes, and CVD are more common in women than men.^4^ Obesity prevalence among men and women aged 25–64 is anticipated to rise by more than 200%, with women having a far higher projected prevalence.^5^ However, cholesterol levels among individuals at 22 years of age can predict CVD development^6^ and prevent CVD by early identification and good lipid treatment, which has great societal value.^7^ The National Cholesterol Education Program (NCEP) recommends hypercholesterolemia testing every five years for adults 20 years or older.^8^ Dyslipidemia is indicated by elevated total cholesterol (TC), LDL-C, or triglycerides or decreased HDL-C.^9^ Atherogenic index of plasma (AIP) which is calculated as the logarithm ratio of plasma triglyceride to HDL content is strongly associated with CVD risk.^10^ AIP accurately predicts coronary heart disease (CHD) by correlating lipoprotein particle size, cholesterol esterification rates, and residual lipoproteinemia. Non-HDL and atherogenic markers were originally considered more essential than LDL concentration for cardiovascular risk management and patient identification.^6^ Wu TT et al.^11^ discovered that AIP predicts CVD risk better than lipid markers.

Some of these figures were highlighted to draw attention to the problem and help us understand its gravity in order to formulate an effective response. In light of this, the current research aimed to assess lipid profile levels and their relationship with anthropometric parameter and AIP in female Taibah University students and employees in Madinah, Saudi Arabia.

## METHODS

A cross-sectional and epidemiological study was performed from January 2019 to January 2020 at Taibah University that involved students and employees. A total of four hundred (n=244) females were recruited that involved both students (n =134) and faculty members (n =110). The targeted demographic was females aged 19-50 years.

### Exclusion Criteria

Women who were pregnant or nursing, those with gastrointestinal tract disease, and chronic diseases such as diabetes, hypertension, or thyroid disorders, were excluded. The study population was selected through systematic random sampling. Initially, the Taibah University official website (www.taibahu.edu.sa) was utilized to collect details about the different colleges, centers, and administration units. Only 244 women agreed to complete the study and did all the blood analysis and anthropometric measurements.

### Anthropometric measurements

An electronic scale (Beurer GmbH Type PS 07, China) was used to measure weight and height twice, while BMI was calculated using formulas (weight (kg) ÷ height(m^2^). BMI categories that applied were; obese ≥ 30, overweight 25-29.9, normal 18.5-25 and underweight < 18.5 [World Health Organization; 2008].^12^

Two measurements were taken of each participant’s waist and hip circumference (WC) using a flexible but non-elastic measuring tape to within half a centimeter. Waist circumference was taken at the level of the natural waist (the narrowest part of the torso) or one finger width under the umbilicus. Hip circumference was determined at the highest circumference of the symphysis pubis anteriorly and the buttocks posteriorly in a parallel plane [Callaway CW].^13^ The waist-to-hip ratio in women was calculated and categorized as low 0.8, moderate 0.8-0.85, and high 0.86, according to WHO [World Health Organization; 2008].^12^

### Specimen Collection and Laboratory Analysis

Fasting blood samples (3ml) were taken from the participants who fasted at least eight hour overnight, to evaluate parameters, involving lipid profile levels. For the purpose of analyzing biomarker profiles, the samples underwent centrifuging at 3000 rpm for five minutes to isolate the serum. serum specimens were kept in a −70°C freezer until analysis. Immunoassay technology (Cobas b 311 immunoassay analyzer) based on the manufacturer’s instructions (Roche Diagnostics, GmbH, Germany) was used to detect the lipid levels. Triglycerides (TG), total cholesterol (TC), HDL-concentration, and LDL-concentration were determined enzymatically [The National Cholesterol Education].^8^ The AIP was determined as log_10_ (TG/HDL-C) as a predictor for CVD. A value < 0.11 for AIP represents a low risk of CVD; values between 0.11-0.21 represent an intermediate risk, but values greater than 0.21 are related to a high risk of CVD [U. I. Nwagha]^14^

### Ethical Approval

The Ethical committee at College of Medicine Taibah University, Madinah, approved this study (IRB 00010413). All participants signed informed consent.

### Statistical analysis

GraphPad Prism seven was used to analyze the data (GraphPad Software, CA, USA). A one-way ANOVA test was used to evaluate quantitative data between subgroupings, and a Pvalue of 0.05 was used to indicate statistical significance. Data were given as mean, standard deviation, or numbers and percentages. The associations between markers were investigated using Pearson’s correlation, and the P-values 0.05 and 0.001 were taken into account as statistically significant.

## RESULTS

The demographic data of the participating females are displayed in Table-I.

**Table-I:** Characteristics of 244 Saudi women participant at Taibah University

Parameter	n= 244 (Mean±SD)	Reference range
Age (years)	24 ±12.5	-
LDL-C (mmol/L)	3.8±1.8	2.59 - 4.11
HDL-C (mmol/L)	1.37±0.3	1.04 - 1.55
Total cholesterol (mmol/L)	6.5±2.0	5.2- 6.2
Triglycerides (TG) (mmol/L)	2.3±0.93	1.7- 2.2
BMI (kg/m^2^)	29.9±5.7	18.5–25.0
Waist-to-hip ratio	0.89±0.7	< 0.8
AIP	0.16±0.1	< 0.1

Values were Geometric Mean ± standard deviation. Bold values were considered abnormal values. References range acquired from Madinah Hospital labs at Madinah region, Saudi Arabia. AIP: atherogenic index of plasma; BMI: body mass index; LDL-C: low-density lipoprotein cholesterol; HDL-C: high-density lipoprotein cholesterol.

### Lipid profile in relation to BMI and AIP

A total of 179 (73.4%) of the participants were overweight and obese, with a mean BMI of 28.79±5.7 kg/m^2^. Overweight and obese women were found to have elevated total cholesterol and triglyceride levels compared to the normal BMI group (all P≤0.05, Table-II). Further to this, the one-way ANOVA multiple comparison analysis demonstrated that women with high BMI (overweight or obese groups) have a high value of the waist-to-hip ratio (≥0.88), whereas the normal BMI group had a low waist-hip ratio (0.73, P=0.02, Table-II).

**Table-II:** Characteristics of participants corresponding to BMI categories.

Parameter	Normal (18.5–25 Kg/m^2^)	Overweight (25.0–29.9Kg/m^2^)	Obese (>30 Kg/m^2^)	P-value

	n=65(26.6%)	n=144(59%)	n=35(14.4%)	
LDL-C (mmol/L)	2.7±1.5	2.8±1.7	2.9±1.6	>0.05
HDL-C (mmol/L)	1.4±0.6	1.2±0.5	1.1±0.7	0.02*
Total cholesterol (mmol/L)	4.6±1.3	5.8±2.8	5.9±2.7	0.003**
Triglycerides (TG) (mmol/L)	1.5±0.74	2.2±1.6	2.8±1.5	<0.001*
Waist-to-hip ratio	0.73±0.5	0.88±0.6	0.89±0.7	0.02*

Data were indicated as the mean ± (SD) for continuous variables, P-value obtained by one-way ANOVA.

* P≤0.05,

** P≤0.00. BMI: body mass index; LDL-C: low-density lipoprotein cholesterol; HDL-C: high-density lipoprotein cholesterol.

The mean lipid profile levels among the women according to AIP categories are shown in Table-III. Out of 244 participants, 120(49.2%) and 44(18%) were at intermediate and high risk of CVD, respectively, as defined by AIP. Females in the intermediate and high-risk groups of CVD had higher levels of lipid profile and higher waist-to-hip ratio compared to the females at low risk(P≤0.001). An exception was the HDL-C level was decreased in the intermediate and high risk CVD groups, compared to the low-risk group based on the AIP index catogries (P=0.001).

**Table-III:** Characteristics of participants corresponding to the AIP index.

Parameter	Low risk < 0.1	Intermediate risk 0.1-0.24	High risk >0.24	P-value

	n=80(32.8%)	n=120(49.2%)	n=44(18%)	
LDL-C (mmol/L)	2.7±1.5	2.8±1.7	3.2±1.2	>0.05
HDL-C (mmol/L)	1.5±0.85	1.2±0.7	1.0±0.5	0.001**
Total cholesterol (mmol/L)	4.8±1.8	5.8±2.2	6.0±2.1	0.001**
Triglycerides (TG) (mmol/L)	1.8±0.64	2.4±1. 4	3.1±1.2	<0.001**
BMI (kg/m2)	24.3±9.86	28.97±10.66	29.6±11.7	0.004**
Waist-to-hip ratio	0.7±0.2	0.86±0.5	0.88±0.7	0.001*

Data were indicated as the mean ± (SD) for continuous variables, P-value obtained from one-way ANOVA.

* P≤s0.05,

** P≤0.001. AIP: atherogenic index of plasma; BMI: body mass index; LDL-C: low-density lipoprotein cholesterol; HDL-C: high-density lipoprotein cholesterol.

There was a positive association between AIP and total cholesterol (r= 0.611), and triglyceride (r= 0.862), but a negative association between AIP and HDL concentration (r= −0.713). However, the BMI had a significantly positive correlation with triglyceride (r= 0.631) and waist-to-hip ratio (r= 0.769). Details of these results are shown in Table-IV.

**Table-IV:** Pearson’s correlation coefficients between AIP, BMI, and Lipid profiles.

Parameter	AIP		BMI	

	r	P	r	P
LDL-C (mmol/L)	0.332	>0.05	0.341	>0.05
HDL-C (mmol/L)	-0.713	0.03*	0.423	>0.05
Total cholesterol (mmol/L)	0.611	0.05*	0.433	>0.05
Triglycerides (TG) (mmol/L)	0.862	0.02*	0.631	0.04*
Waist-to-hip ratio	0.311	>0.05	0.769	0.03*

P-values were obtained from Pearson's correlation; Starred values point to a significant level

* P≤0.05,**P≤0.00. AIP: atherogenic index of plasma; BMI: body mass index; LDL-C: low-density lipoprotein cholesterol; HDL-C: high-density lipoprotein cholesterol.

For participants aged between 41 and 50, a greater BMI was associated with a greater AIP, which was statistically different (P<0.05) from other research groups that were divided by age group. Table-V gives additional information.

**Table-V:** Characteristics of participants’ BMI and AIP corresponding to age groups.

Age (19-50 years)	BMI (18.5 - 25.0 kg/m^2^)	AIP (<0.11)
19-20	23.6±4.4	0.05±0.01
21-30	23.7±4.6	0.05±0.01
31-40	26.5±7.8	0.11±0.11
41-50	28.5±8.4*	0.13±0.11*

Values were Geometric Mean ± standard errors. AIP: atherogenic index of plasma; BMI: body mass index. Starred values show a significant level

* P≤0.05.

## DISCUSSION

We used a large dataset consisting of Taibah University female students and staff members to analyze the association between lipid profile levels and anthropometric variables and AIP in females at Taibah University in Madinah, Saudi Arabia.

In the present study, participants had abnormal lipids, overweight and obese women were found to have elevated total cholesterol (≥5.8), and triglyceride (≥2.2) levels, and the excessive levels of these lipids and the abnormal balance between them increase the risk of cardiovascular events, such as heart attack and stroke.^15^ Our findings also revealed that there was a non-significant difference (p>0.05) between normal, overweight, and obese participants in terms of LDL and HDL concentration, while there was a significant difference(P≤0.05, 0.001) in terms of TC and TG. In addition, there was an increase in AIP with age (minimum=0.05 for 19-20 age group and maximum=0.13 for 41-50 age group) which clearly indicates that with increase in age there was an eioght fold increase in hyper cholesterolemia, which is well supported by a study that found participants aged 40–49 have a 4.5 increased incidence of hypercholesterolemia, compared to those aged 20 and younger. In individuals aged 30-39, hypercholesterolemia was three times more prevalent.^14^ This information is consistent with our findings, which indicated that the highest value of AIP occurred in older females (41-50) and increased progressively with age.

The reason for this abnormal lipid level is the excess weight itself, and a diet containing a high level of fats. Obesity prevalence in Saudi Arabia is high: 30.4% of males and 10.2% of females aged 18-29 are overweight or obese (BMI 25 kg/m^2^).^16^ Furthermore, 30% of Saudi Arabian adolescents are either overweight (14.1%) or obese (15.9%)^17^, conditions associated with CVD and death. This is well supported by the findings of a study that showed how in Saudi Arabia, BMI may be the single most important risk factor for death. In 2017, high BMI was directly responsible for more than a quarter of all fatalities from non-communicable diseases (NCDs). The high rates of obesity and dyslipidemia, which put people at greater risk for CVD, were reflected in this study’s AIP index. In the present study, there were abnormal values for TC (6.5±2.0), TG (2.3±0.93), BMI (29.9±5.7), and waist to hip ratio (0.89±0.7), which clearly indicated dyslipidemia.

According to a survey that was conducted in Saudi Arabia in 2022 by AlMuhaidib et al.,^18^ researchers discovered that approximately one quarter of Saudi adolescents (33.3% of males and 17.9% of females) had dyslipidemia. There was a statistically significant difference (p<0.001) between the regions as TC was found to be 6.7% of the population, LDL concentration was found to be 7.1%, HDL concentration was found to be 12.8%, non-HDL concentrations were found to be 8.3%, and triglycerides were found to be 9.6%. Both BMI (OR = 0.80, 95% CI Confidence Interval) ranging from 0.69 to 0.94) and age were found to be independently associated with dyslipidemia.^19^ In contrast to our investigation and determination of the lipid profile alterations in females ranging from 19 to 50 years of age, theirs showed the factors linked with dyslipidemia only in Saudi adolescents aged 10 to 19 years old.^19^ Differences in the general frequency of dyslipidemia and the proportion of abnormal lipid levels between studies may be due to changes in methods (age group, lipid cutoff reference, or fasting state), lifestyle variables like nutritional habits, demographic characteristics, genetic background, and the time gap between studies.

In the present study, waist-hip ratio WHR was also found to be abnormal (0.89±0.7) with AIP (0.16±0.1) in participants. ]. WHR it is not a direct measure of the proportion of body fat, but rather a composite measure of fat mass and fat-free tissue mass; the adipose component of WHR is small, particularly in nonobese subjects Increased waist-hip ratio may be a sign of higher waist circumference (reflecting increased visceral fat), reduced hip circumference (reflecting low gluteal muscle mass and/or low peripheral fat mass), or a combination.

This means waist circumference and AIP are related in some way, which is well supported by a study conducted in Iran which revealed that the AIP value was shown to be favorably related to waist circumference.^20^

In the present study, a normal value for LDL was found among the participants. These findings were in contrast to a study where it was found that 8.2% of women had cholesterol levels of >6.2 mmol/L, in a community-based investigation. Additionally, it has also been confirmed that the rate of hypercholesterolemia increases with participants’ ages.^21^ The frequency of hypercholesterolemia among Saudi Arabia’s general population and among stable clinic visitors varies substantially across the country, from 8.5% to 54.9%.^22^ A different study found that 12.5% of the sample was affected by hypercholesterolemia. The prevalence of hypercholesterolemia increased moderately with age (r=0.240, P<0.0001), suggesting that old age is correlated with a higher risk of developing hypercholesterolemia.^23^ The current investigation found 120 participants (49.2%) and 44 participants (18%) classified as having an intermediate and a high risk of CVD. The lipid profile and waist-to-hip ratio of women in the intermediate and high-risk groups for CVD were significantly greater than those of women in the low-risk group (P≤0.05). In contrast, no significant difference was found between the prevalence of dyslipidaemia in men (72.6%) and women (72.3%) in the Northern Emirates local adult population, which was 72.5% (95% CI 69.3- 75.4). Low HDL concentration was more common among females (47.4%, 60.2%) than males (39%, 27.9%) (p=0.006).^24^ However, approximately 28.5 million people (>20 years) have high total serum cholesterol and a reported prevalence of 11.9%.^25^

Moreover, the prevalence of hypercholesterolemia among Saudis is 8.5%, according to previously published studies with high sample sizes.^26^ Meanwhile, another study reported that one in four people in Saudi Arabia had high TC ( > 200 mg/dl), high triglycerides (> 150 mg/dl), high LDL concentration (> 130 mg/dl), and low HDL concentration (40 mg/dl) while 60% of the population had dyslipidemia.^27^ It is common knowledge that elevated triglyceride levels raise the risk of CVD by elevating the proportion of LDL. This is because atherogenic tiny dense LDL particles can speed up to generate reactive oxygen species and lipid peroxidation, leading to the development of atherosclerosis. Meanwhile, men in Saudi Arabia had a higher 10-year high/intermediate risk of CVD than women; however, this risk rises with age for both sexes (33.1% versus 17.1%)^4^ As seen in the present study, AIP increases with age: at age 31-40 AIP was 0.11±0.11, and at 41-50 it was 0.13±0.11 while at the age of 19-20 and 21-30 years, it was 0.05±0.01.

BMI also increases with age, which clearly indicates that with the increase in age, risk of CVD also increases. Our findings are in line with a study in which researchers concluded that age, BMI, in addition to unhealthy diet, smoking, blood pressure, and family history of CVD were independently connected to CVD risk.^27^ In the present study, AIP was compared with triglycerides and HDL, which is recognized as an important cardiometabolic risk factor and is gaining popularity as a screening tool for dyslipidemia.^20^ A positive association between AIP and total cholesterol (r= 0.611), and between AIP and triglycerides (r= 0.862) was found, but a negative association between AIP and HDL cholesterol (r= 0.713) was found. This is due to the fact that AIP has an inverse relationship with LDL particle size and a positive relationship with the HDL fractional esterification rate (FERHDL). This ratio is a sensitive predictor of coronary atherosclerosis and cardiovascular risk because it accurately reflects the presence of atherogenic small LDL and HDL particles. Our findings are in line with the findings of a study in which there was a negative link between AIP and HDL cholesterol and a positive correlation with triglycerides, total cholesterol and LDL cholesterol.^20^ Meanwhile, some Saudis may find it challenging to maintain an active lifestyle due to environmental and cultural barriers, a lack of convenient access to fitness centers, and a general absence of these resources.^26^,^27^ In Saudi Arabia overweight adults spend more than nine hours each day in front of electronic media. Multiple-device social media use, video game playing, mobile and/or online gaming, television viewing, and multitasking all contribute to the average person’s daily screen time. In addition to this, emotional eating, or binge eating in response to negative emotions like stress, anxiety, or depression, is another problematic eating behavior that is highly associated with a higher BMI. This study linked anthropometric characteristics to lipid profile levels to enhance CDV prevention measures. Healthy living is the most effective approach to decrease cholesterol and CHD risk. Walking to and from school or work instead of driving may increase physical activity. Walking, biking, or taking public transit to school boosts energy and physical exercise. Psychological treatment is recommended by the Saudi Arabia MoH for weight control programs to assist patients cut calories, exercise more, and reduce inactive time. The National Transformation Program and Vision 2030 passed laws allowing public school females to pursue physical exercise classes. Saudi Vision 2030 included the “Healthy Food Strategy” from the Saudi Food and Drug Administration. This approach promotes healthy lifestyles and reduces sugar, salt, and fat intake through dietary adjustments and education. The National Transformation Program has been implemented to improve healthcare quality The government also prioritizes primary CVD prevention and large quality-of-life projects.

### Limitaions

There are some limitations to this study, such as the small sample size and the choice to focus solely on college females to prevent extrapolation to the wider populace. Since this was a cross-sectional analysis, no causal link can be drawn. However, dyslipidemia is common among Saudi women aged 19-50 years. This is the first research in Madinah city of its kind to create a full image of the lipid profile panel and the factors associated with it, such as BMI and AIP.

## CONCLUSION

Total cholesterol and triglyceride levels were found to be abnormally high in the present study’s group of overweight and obese women. The AIP had a positive correlation with total cholesterol, triglycerides, and a negative correlation with HDL cholesterol. While AIP is a reliable prediction of future atherosclerosis and CVD, it was found to have a substantial positive connection with total cholesterol and triglyceride, both of which suggested risk of CVD. Furthermore, the government has previously acknowledged the importance of primary prevention of CVD disorders and approved four significant projects aimed at enhancing lifestyles. A multidisciplinary team must take action to educate the public and to disseminate knowledge on CVD risk and prevention to a wider audience.

### Key Findings:

- Most of the subjects were either overweight or obese.
- They had elevated triglyceride and total cholesterol levels; and they had an excessively high waist-to-hip ratio.
- The AIP was found to have a significant positive correlation with total cholesterol and triglycerides, both of which suggested a risk of CVD, and a negative correlation with HDL cholesterol.
- AIP is a reliable predictor of future atherosclerosis and CVD.
- Overweight and obese women are an underserved population, and a multidisciplinary team is needed to raise awareness about cardiovascular disease risk and prevention.

### Author`s Contribution:

**AMQS:** Literature search, provided research materials, collected and organized data and references, and provided logistic support, and wrote the final draft of the article.

**WM:** Labratory study and data anaylsis. Identified the appropriate methods of analysis, interpreted the results of study.

**EMA:** Conceptualized the idea of research helped in enriching references.

The authors engaged in comprehensive discussions regarding the outcomes and made significant contributions to the final manuscript. They assumed responsibility and were held accountable for ensuring the precision and integrity of the research.
